# Numerical Analysis of Keyhole and Weld Pool Behaviors in Ultrasonic-Assisted Plasma Arc Welding Process

**DOI:** 10.3390/ma14030703

**Published:** 2021-02-02

**Authors:** Junnan Qiao, Chuansong Wu, Yongfeng Li

**Affiliations:** MOE Key Lab for Liquid-Solid Structure Evolution and Materials Processing, Institute of Materials Joining, Shandong University, Jinan 250061, China; qjnqjn@yeah.net (J.Q.); lyf452433605@126.com (Y.L.)

**Keywords:** plasma arc welding, acoustic radiation force, plasma arc pressure, gas shear stress, weld pool, keyhole behavior

## Abstract

The acoustic radiation force driving the plasma jet and the ultrasound reflection at the plasma arc-weld pool interface are considered to modify the formulas of gas shear stress and plasma arc pressure on the anode surface in ultrasonic-assisted plasma arc welding (U-PAW). A transient model taking into account the dynamic changes of heat flux, gas shear stress, and arc pressure on the keyhole wall is developed. The keyhole and weld pool behaviors are numerically simulated to predict the heat transfer and fluid flow in the weld pool and dynamic keyhole evolution process. The model is experimentally validated. The simulation results show that the acoustic radiation force increases the plasma arc velocity, and then increases both the plasma arc pressure and the gas shear stress on the keyhole wall, so that the keyholing capability is enhanced in U-PAW.

## 1. Introduction

**Figure 1:**
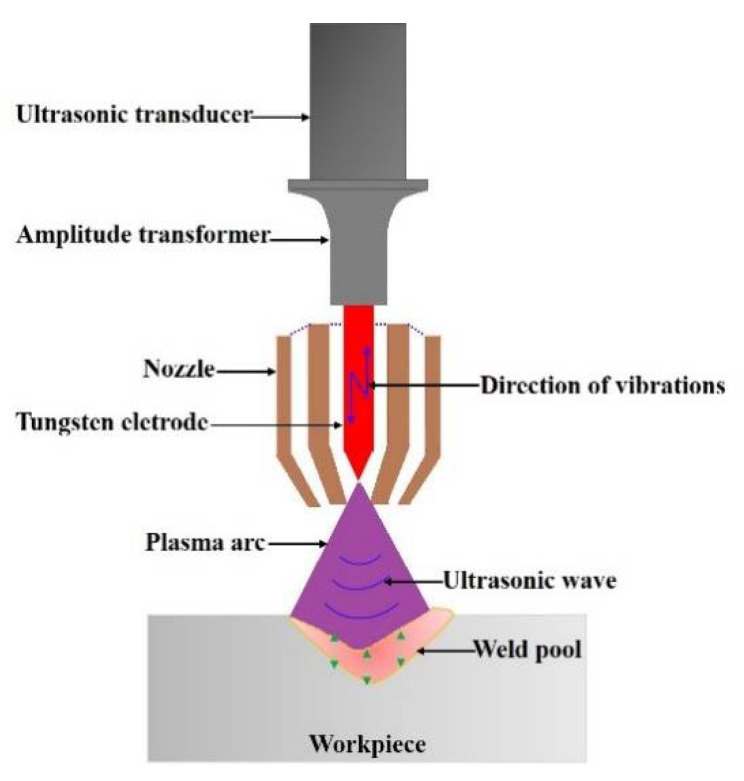
Schematic illustration of the ultrasonic-assisted plasma arc welding process.

Plasma arc welding (PAW) can form a keyhole channel inside the weld pool along the plate thickness, and join materials of mid-thickness in a single pass [1]. The stability of the weld pool and keyhole is the prerequisite for obtaining a large penetration and good weld quality [2]. However, in the PAW process, the dynamic stability of the weld pool and keyhole is poor, and the welding process-parameter window is narrow, which restricts its wider applications in the industry [3,4]. To further improve the weld pool dynamic stability and the keyholing capability in PAW, Wu et al. developed a process variant of PAW, i.e., ultrasonic-assisted plasma arc welding (U-PAW) [5], which exerts ultrasonic vibration on the tungsten electrode, as schematically illustrated in Figure 1. Different from ultrasonic-assisted TIG welding and ultrasonic-assisted MIG welding [6,7], due to the special structure of plasma arc welding torch and the setback of the tungsten electrode, a U-PAW torch was designed. The ultrasonic vibration at the end of the tungsten electrode is directly transmitted into the plasma arc, leading to the enhancement of heat-pressure characteristics, therefore the keyholing capability of plasma arc can be improved [8,9].

To study the process mechanism of U-PAW, Wu et al. [5] conducted experiments under different welding process parameters, and found that by the application of ultrasonic vibration, the plasma arc pressure is increased, so that an open keyhole can be formed at a higher welding velocity or lower welding current in U-PAW. The current density on the anode surface in U-PAW is increased due to the further constricted arc column [8]. Wang et al. [9] used the controlled pulse current waveform in U-PAW and carried out the closed-loop experiments. They found that with the application of ultrasonic vibration, the peak level of the welding current for a specific steel plate thickness is decreased. The above experimental results show that the ultrasonic vibration improves the keyholing ability in U-PAW and broadens the welding process window. An experimental investigation can only provide the test data for specific welding conditions, while the analytical method and numerical simulation can reveal the underlying physical mechanism in the welding process. However, the analytical formulae for thermal conduction in welding was derived based on some unreasonable assumptions for simplification, and large errors exist when they are used to predict the temperature distribution near and in the weld pool [10]. On the other hand, although the analytical method can save the calculation time [11,12], it can only be used to calculate the temperature field but not the flow field in the molten pool. Therefore, it is essential to conduct numerical modeling and simulation of fluid flow and heat transfer in the molten pool in both PAW and U-PAW for a complete understanding of the U-PAW process mechanism. 

Recently, many researchers have conducted a numerical analysis of weld pool and keyhole behaviors in PAW [13,14,15,16,17,18,19]. However, there is little such work on the newly developed U-PAW. Li et al. [20,21] developed a model to study the interaction between the ultrasonic vibration and plasma arc from macroscopic and microscopic aspects, but did not consider the influence of plasma arc on the weld pool. When the plasma jet collides the workpiece surface, it will produce a stagnation pressure, called the arc pressure. According to the interaction between the ultrasonic and plasma arcs, the author deduced the arc pressure model [22]. However, the arc pressure was determined by assuming that the acoustic energy was totally absorbed by the plasma arc. 

In this study, a mathematical model for weld pool and keyhole behaviors in U-PAW is developed. The arc pressure and gas shear stress formulae are modified by considering the acoustic radiation force and the ultrasonic reflection at the plasma arc-weld pool interface. The effects of the arc pressure and gas shear stress on the fluid flow and keyhole behavior in the weld pool were analyzed quantitatively, and the numerical simulation results are verified experimentally.

## 2. Formula of Plasma Arc Pressure in U-PAW

An ultrasonic action on the plasma arc medium will produce the corresponding acoustic radiation force, which is directly proportional to the sound intensity [23]. Based on the acoustic impedance theory, the interface between the plasma arc and weld pool can be taken as a hard boundary. The total ultrasound reflection occurs at the interface between the plasma arc and weld pool. The difference of the acoustic radiation force at the end of the tungsten electrode and the anode surface, $\bar{F}$, is written as follows [24]:

(1) $$\bar{F}=\frac{2I_{0}S_{t}}{c}\left(1-e^{-2\alpha L}\right)$$

where $I_{0}$ is the sound intensity, $S_{t}$ is the area of tungsten end, c is the sound speed, L is the arc length, and α is the attenuation coefficient of sound pressure in the plasma arc.

In the plasma arc, during time interval Δt, the acoustic radiation force will do some work equal to $\bar{F}c\Delta t$, while the electric field will do some work equal to $\eta_{i}IU\Delta t$, where $\eta_{i}$ is the conversion efficiency [15], I is the welding current, and U is the arc voltage. According to the kinetic energy theorem, we obtain the following:

(2) $$\bar{F}c\Delta t+\eta_{i}IU\Delta t=\frac{1}{2}mv_{2}^{2}-\frac{1}{2}mv_{1}^{2}$$

(3) $$m=\rho_{\mathrm{Ar}}Q_{\mathrm{plas}}\Delta t$$

where m is equal to the mass of the plasma gas ejected from the nozzle exit within the time interval Δt, $v_{1}$ is the velocity of the plasma gas at the nozzle exit, $v_{2}$ is the velocity of the plasma arc near the anode surface, $\rho_{\mathrm{Ar}}$ is the plasma gas density, and $Q_{\mathrm{plas}}$ is the plasma gas flow rate.

Since the vibration of the tungsten end is harmonic vibration, the amplitude of the sound pressure is $p_{u}=\rho_{\mathrm{Ar}}cv_{u}$, and the amplitude of the vibration velocity is $v_{u}=2A\pi f$, where A is the vibration amplitude, and f is the frequency. Therefore,

(4) $$I_{0}=\frac{p_{u}}{\sqrt{2}}\cdot \frac{v_{u}}{\sqrt{2}}=\frac{1}{2}\left(\rho_{Ar}cv_{u}\right)v_{u}=\frac{1}{2}\rho_{Ar}c{\left(2\pi Af\right)}^{2}=2\pi^{2}A^{2}f^{2}c\rho_{Ar}$$

Combined with Equations (1)–(4), we get the following:

(5) $$v_{2}^{2}-v_{1}^{2}=\frac{2\bar{F}c+2\eta_{i}IU}{\rho_{Ar}Q_{plas}}$$

Based on Equations (1) and (4), we get:

(6) $$\bar{F}c=2I_{0}S_{t}\left(1-e^{-2\alpha L}\right)=4\pi^{2}A^{2}f^{2}c\rho_{Ar}S_{t}\left(1-e^{-2\alpha L}\right)$$

(7) $$v_{1}=\frac{Q_{plas}}{S_{noz}}$$

where $S_{noz}$ is the nozzle exit area. 

Manipulating Equations (5)–(7), we can get:

(8) $$v_{2}=\sqrt{\frac{8cA^{2}\pi^{2}f^{2}S_{t}\left(1-e^{-2\alpha L}\right)}{Q_{plas}}+\frac{2\eta_{i}IU}{\rho_{Ar}Q_{plas}}+{\left(\frac{Q_{plas}}{S_{noz}}\right)}^{2}}$$

The plasma arc velocity $v_{2}$ produces pressure on the surface of the anode. The arc pressure is assumed in the Gaussian distribution on the anode surface. In addition, considering the action of its own magnetic field inside the plasma arc, a coefficient $\beta_{M}$ is introduced as follows:

(9) $$\beta_{M}=\frac{\mu_{0}I^{2}}{4\pi^{2}}\cdot \frac{1}{A_{I}}$$

where $\mu_{0}$ is the permeability in a vacuum, and $A_{I}$ is a constant.

With the modified plasma arc velocity $v_{2}$ described by Equation (8), the similar derivation procedure in [22] may be used for the next step, and not iterated here. The modified arc pressure formula is written as follows:

(10) $$\begin{array}{ll} P\left(x,y\right)= & 3\beta_{M}\frac{r_{noz}^{2}}{r_{p}^{2}}\rho_{Ar}\exp\left(-\frac{3\left(x^{2}+y^{2}\right)}{r_{p}^{2}}\right)\\ & \sqrt{\frac{8\rho_{Ar}cA^{2}\pi^{2}f^{2}S_{t}\left(1-e^{-2\alpha L}\right)Q_{plas}+2\eta_{i}IUQ_{plas}}{\rho_{Ar}S_{noz}^{2}}+{\left(\frac{Q_{plas}}{S_{noz}}\right)}^{4}} \end{array}$$

where $r_{P}$ is the radius of the plasma arc pressure, $r_{noz}$ is the nozzle exit radius, and (*x*,*y*) is the coordinate away from the torch axis.

Inside the keyhole, the keyhole radius is gradually smaller from the top to the bottom surface of the workpiece, which means that the keyhole channel further shrinks the plasma arc [25]. Therefore, the plasma arc pressure inside the keyhole increases with the keyhole depth, and a coefficient $\chi_{p}$ is introduced to describe this effect as follows:

(11) $$\chi_{p}=1+\frac{\Gamma \left(x,y\right)}{ZL+H_{nw}}$$

where Γ(x,y)=z is the function of the keyhole wall, $H_{nw}$ is the distance between the nozzle exit and the top of the workpiece surface, and ZL is the thickness of the workpiece. Finally, the plasma arc pressure is written as follows:

(12) $$\begin{array}{ll} P\left(x,y,z\right)= & 3\chi_{p}\beta_{M}\frac{r_{noz}^{2}}{r_{p}^{2}}\rho_{Ar}\exp\left(-\frac{3\left(x^{2}+y^{2}\right)}{r_{p}^{2}}\right)\\ & \sqrt{\frac{8\rho_{Ar}cA^{2}\pi^{2}f^{2}S_{t}\left(1-e^{-2\alpha L}\right)Q_{plas}+2\eta_{i}IUQ_{plas}}{\rho_{Ar}S_{noz}^{2}}+{\left(\frac{Q_{plas}}{S_{noz}}\right)}^{4}} \end{array}$$

**Figure 2:**
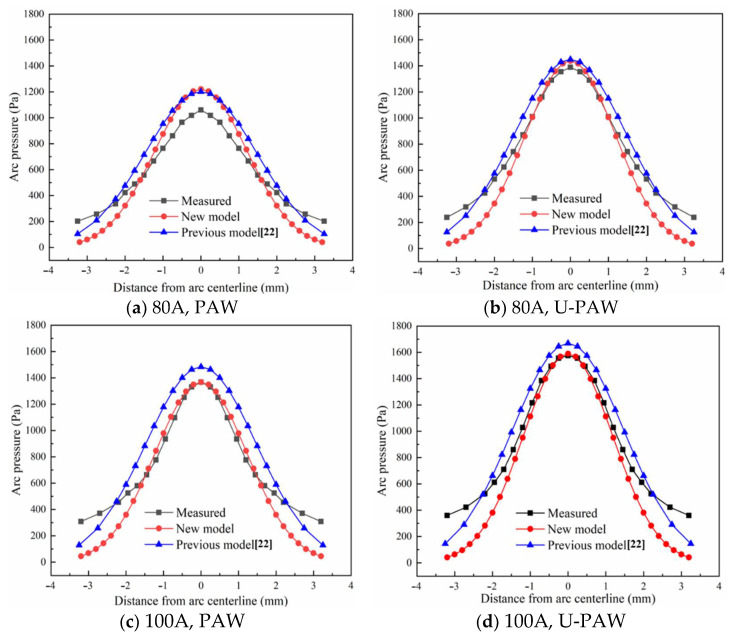
Plasma arc pressure distribution.

Equation (12) includes the contribution components of the plasma arc pressure in U-PAW, including the acoustic effect term, electric field term, and plasma gas flow rate term. The early studies only correlated the arc pressure with the square of the welding current [26,27]. Though Li et al. [28] and Lang et al. [29] proposed other factors such as density, the nozzle exit area, and plasma gas flow rate, their formulae were suitable for the conventional PAW. Equation (12) is for the U-PAW process. The plasma arc pressure was measured by the water-cooled copper anode method. The detailed measurement method may be found in [5]. According to the calculation results in the literature [30], the arc pressure drops to a very small value at *r* = 3 mm, so the plasma arc pressure action radius $r_{P}$ is taken as 3 mm. For the plasma arc pressure on the anode surface, $\chi_{p}=1$. Under the welding conditions listed in Table 1, the distribution of PAW and U-PAW arc pressure was calculated by Equation (12), as shown in Figure 2. Compared with the previous formula [22], the arc pressure distribution predicted by the modified formula is more consistent with the experimental measurements. It can be seen that the arc pressure in U-PAW is greater than that in PAW. Therefore, Equation (12) is more reasonable.

## 3. Gas Shear Stress Formula

In the literature, the maximum value of the wall gas shear stress produced by the vertical jet is expressed as follows [31]:

(13) $$\tau ={\left(\frac{\rho_{Ar}\mu }{D}\right)}^{\frac{1}{2}}{\left(\frac{D}{L}\right)}^{2}v_{2}^{1.5}$$

where μ is the plasma arc viscosity, D is the nozzle diameter, L is the distance between the nozzle exit and the top of the workpiece surface, and $v_{2}$ is the plasma arc velocity.

The plasma arc velocity $v_{2}$ at the workpiece surface is described by Equation (8). The maximum value of the plasma arc gas shear stress is written as follows:

(14) $$\tau_{u}={\left(\frac{\rho_{Ar}\mu }{D}\right)}^{\frac{1}{2}}{\left(\frac{D}{L}\right)}^{2}\chi_{p}^{1.5}{\left(\frac{8\rho_{Ar}cA^{2}\pi^{2}f^{2}S_{t}\left(1-e^{-2\alpha L}\right)+2\eta_{i}IU}{\rho_{Ar}Q_{plas}}+{\left(\frac{Q_{plas}}{S_{noz}}\right)}^{2}\right)}^{0.75}$$

For PAW without the ultrasonic vibration, the values of *A* and *F* are zero, so that Equation (14) can also be used to calculate the gas shear stress in PAW. Meng et al. [32] showed that the plasma shear stress increases linearly near the center of the weld pool surface, and then decreases rapidly. According to the results of the gas shear stress described by Wu et al. [18], its dimensionless distribution function is as follows:

(15) $$g(r)=\{\begin{array}{l} 0.18r^{4}-0.95r^{3}+1.37r^{2}+0.0957\;\;\;\;\;\;(r\leq 0.208\;mm)\;\\ 1.896\exp(-0.315r)\;+0.022\;\;\;\;\;(r>0.208\;mm)\;\;\; \end{array}$$

where $r=\sqrt{x^{2}+y^{2}+z^{2}}$.

Therefore, the plasma arc gas shear stress formula is as follows:

(16) $$\tau_{u}\left(x,y,z\right)=\tau_{u}g\left(r\right)$$

**Figure 3:**
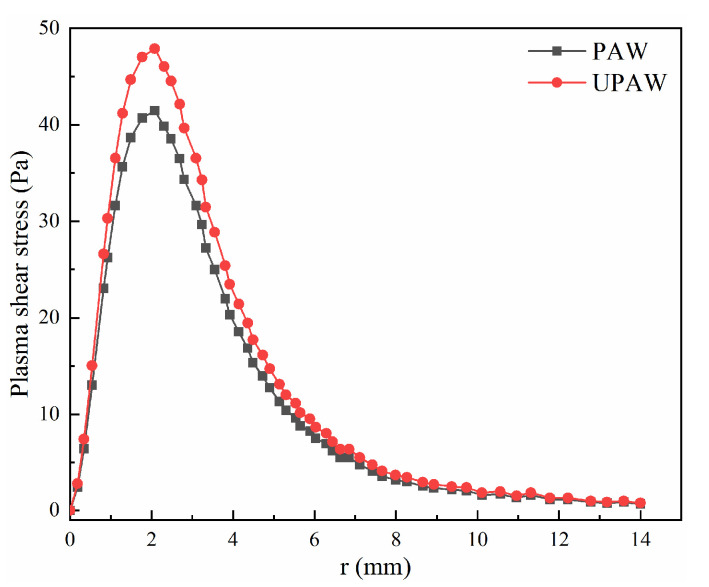
Gas shear stress distribution (welding current 80 A).

Under the conditions listed in Table 1, the calculated gas shear stress on the anode surface are shown in Figure 3. It can be seen that after the ultrasound application, the plasma arc velocity increases, and then the peak value of the gas shear stress on the workpiece surface increases, and the peak value of the gas shear stress is 2 mm away from the torch axis, which is different from the result in the literature [18,19]. In this paper, the nozzle exit radius is 3.2 mm, so the arc pressure and gas shear stress are small. In the variable polarity PAW, with a peak welding current of 150 A, the peak value of the gas shear stress on the workpiece surface was about 85 Pa [33]. Bai et al. [34] gave a peak gas shear stress of 80 Pa when the welding current was 169 A. Therefore, the calculated value of the gas shear stress is reasonable. 

Equation (16) gives the shear stress on the workpiece surface. In PAW, the weld pool surface is concave and deformed, so that the gas shear stress distribution will change with the keyhole shape. According to the description in the literature [32], when the weld pool surface is concave and deformed, there is a following relation between the plasma gas shear stress vector $\vec{\tau }$, the normal vector $\vec{n}$ of the deformed weld pool surface, and the gas shear stress acting direction vector $\vec{o}=\left(x,y,0\right)$ before the deformation of the weld pool surface, as follows:

(17) $$\left\{ \begin{array}{l} \vec{\tau }\cdot \vec{n}=0\\ \left|\vec{\tau }\right|=\tau \left(x,y,z\right)\\ \vec{\tau }=A\vec{o}+B\vec{n} \end{array}\right.$$

where A and B are arbitrary real numbers.

## 4. Model of the Weld Pool and Keyhole 

**Figure 4:**
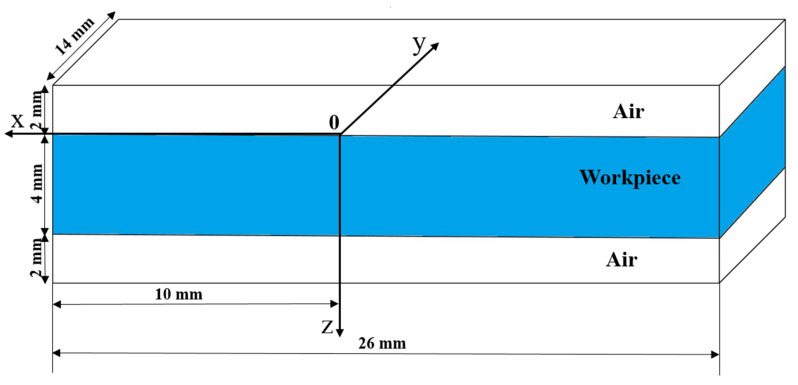
Calculation domain.

Owing to the symmetry of the workpiece, half of the calculation domain is used in the molten pool simulation. As shown in Figure 4, the size of the calculation domain is 26 mm × 14 mm × 10 mm. The thickness of the workpiece is 4 mm, and the air layer is 2 mm above and below the plate. The grid is evenly divided with a size of 0.2 mm. In this model, the welding torch is stationary while the workpiece is moving. The Cartesian coordinate system is established by taking the intersection of the center axis line of the welding torch and the upper surface of the workpiece as the coordinate origin.

Due to the interaction between the ultrasound, weld pool, and plasma arc in the U-PAW process, the physical phenomena involved are very complicated. The following simplifications and assumptions have to be made in the current research: (1) In the U-PAW process, since the density and sound velocity in liquid metal are relatively high, the acoustic streaming driving force caused by the difference of the acoustic energy density can be neglected. Therefore, the influence of sound pressure on the molten metal flow and heat transfer is temporarily not considered; (2) the weld pool flow is incompressible and laminar. Thereby, the heat transfer and fluid flow in the molten pool can be described by following governing equations.

Continuity equation:

(18) $$\frac{\partial \rho }{\partial t}+\nabla \cdot \left(\rho \vec{v}\right)=0$$

where ρ is the density, t is the time, and $\vec{v}$ is the fluid velocity vector.

Momentum equation:

(19) $$\frac{\partial }{\partial t}\left(\rho v\right)+\nabla \cdot \left(\rho v\vec{v}\right)=-\nabla p+\mu \nabla^{2}v+F_{v}$$

where p is the pressure in fluid, μ is viscosity, $F_{v}$ is the momentum source term.

Energy equation:

(20) $$\frac{\partial }{\partial t}\left(\rho H\right)+\nabla \cdot \left(\vec{v}\rho H\right)=\nabla \cdot \left(k\nabla T\right)+Q_{v}$$

where H is the enthalpy, k is the thermal conductivity, *T* is the temperature, and $Q_{v}$ is the source term of the energy equation.

VOF equation:

(21) $$\frac{\partial }{\partial t}\left(\phi \right)+\nabla \cdot \left(\phi \vec{v_{s}}\right)=0$$

(22) 0<ϕ<1

According to the description in [15], Equation (21) cannot accurately describe the interface of the plasma arc and the molten pool, so that a function δ is introduced to describe the gas-liquid interface and describe the physical interface definition function $\delta_{1}$ of the upper surface of the workpiece:

(23) $$\delta_{1}=\left\{ \begin{array}{ll} 1 & \mathrm{if}\;\phi \geq \varepsilon_{1}\;\mathrm{and}\;\left|\nabla \phi \right|\geq \varepsilon_{2}\;\mathrm{and}\;\phi_{z}\geq \varepsilon_{3}\;\\ 0 & \mathrm{else}\; \end{array}\right.$$

The physical interface defining function $\delta_{2}$ describing the upper and lower surfaces of the workpiece is as follows:

(24) $$\delta_{2}=\left\{ \begin{array}{ll} 1 & \mathrm{if}\;\phi \geq \varepsilon_{1}\;\mathrm{and}\;\left|\nabla \phi \right|\geq \varepsilon_{2}\\ 0 & \mathrm{else}\; \end{array}\right.$$

where $\varepsilon_{1}$, $\varepsilon_{2}$, $\varepsilon_{3}$ are very small constants. 

The heat source, surface tension, arc pressure, electromagnetic force, gas shear stress, buoyancy, and the resistance force in a mushy zone are taken as the boundary conditions of the weld pool. The treatment method of the heat source model and arc pressure at the plasma arc-weld pool interface has been described in detail in our previous paper [22]. 

Table 2 lists two study cases. The thermophysical properties of a 304 stainless steel can be obtained from our previous work [22]. The boundary conditions are written by the user defined function (UDF), and are brought into the source terms of energy and momentum conservation equations to be solved. The equations are discretized by the finite volume method (FVM). The governing equations and boundary conditions were solved by the numerical method.

## 5. Experimental Process

**Figure 5:**
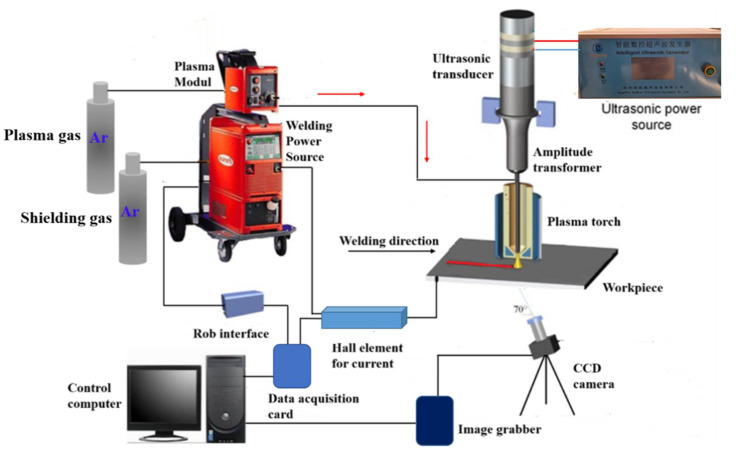
Schematic illustration of the ultrasonic-assisted plasma arc welding (U-PAW) process setup.

**Figure 6:**
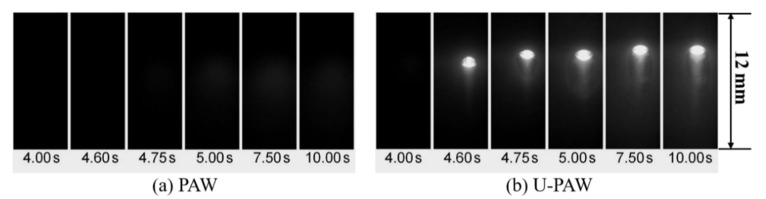
The captured images of the keyhole exit (**a**) PAW (**b**) U-PAW.

Figure 5 is a schematic diagram of the U-PAW welding process setup. A vision system was developed to observe the keyhole exit and keyholing time from the backside of the workpiece [35]. As shown in Figure 6a, no open keyhole was detected in PAW. For U-PAW, Figure 6b shows that an open keyhole was firstly monitored at an instant of 4.6 s. 

## 6. Results and Discussion

**Figure 7:**
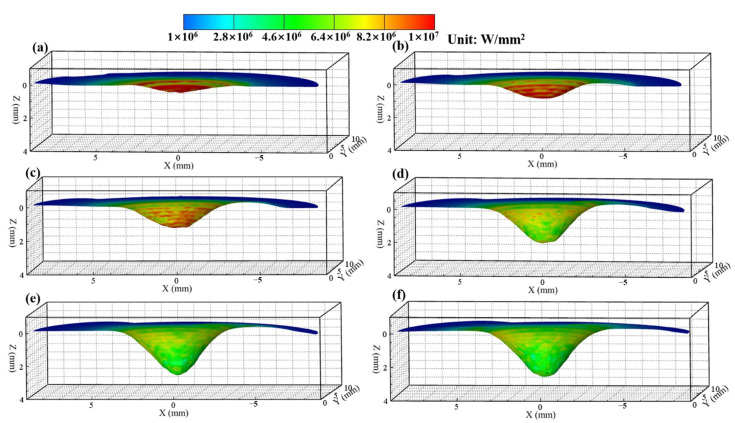
The dynamic keyhole evolution process in PAW (**a**) 1.21, (**b**) 2.02, (**c**) 2.5, (**d**) 3.2, (**e**) 4.01, (**f**) 4.6 s.

**Figure 8:**
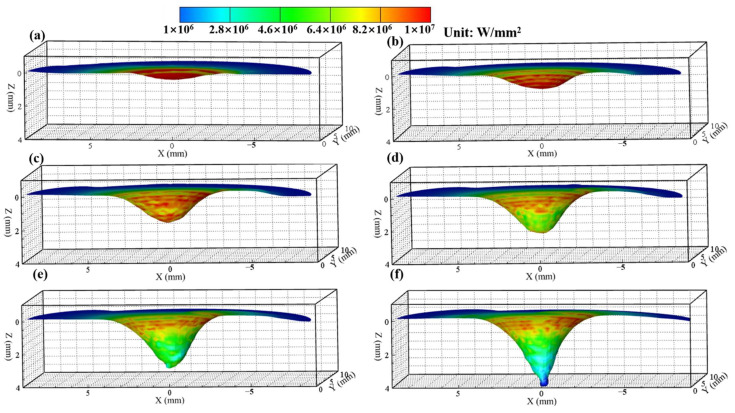
The dynamic keyhole evolution process in U-PAW (**a**) 1, (**b**) 2.01, (**c**) 2.6, (**d**) 3, (**e**) 3.31, (**f**) 4.2 s.

Figure 7 and Figure 8 show the calculated keyhole dynamic evolution process in PAW and U-PAW, respectively. At the initial time, the peak heat flux is mainly distributed at the top part of the keyhole wall. With the increase of the keyhole depth, the consumed plasma arc heat gets larger, the heat flux at the bottom part of the keyhole is lower, and the heat flux at the bottom part of the keyhole is smaller than that at the top part. As shown in Figure 7e, at t = 4.01 s, the gas shear force, Marangoni shear force, and arc pressure on the keyhole wall reach equilibrium. Therefore, the keyhole depth does not change any more in PAW. However, in U-PAW, the larger arc pressure promotes the keyhole formation, and the bottom of the keyhole continuously transmits the plasma arc heat flow and arc pressure to the weld pool. As shown in Figure 8f, at t = 4.2 s, an open keyhole is formed.

**Figure 9:**
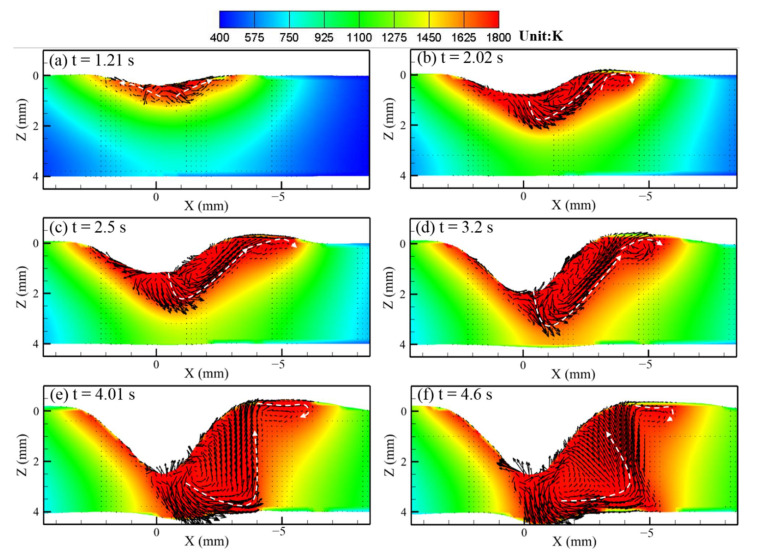
The calculated temperature field, fluid flow, and keyholing time in the PAW case.

**Figure 10:**
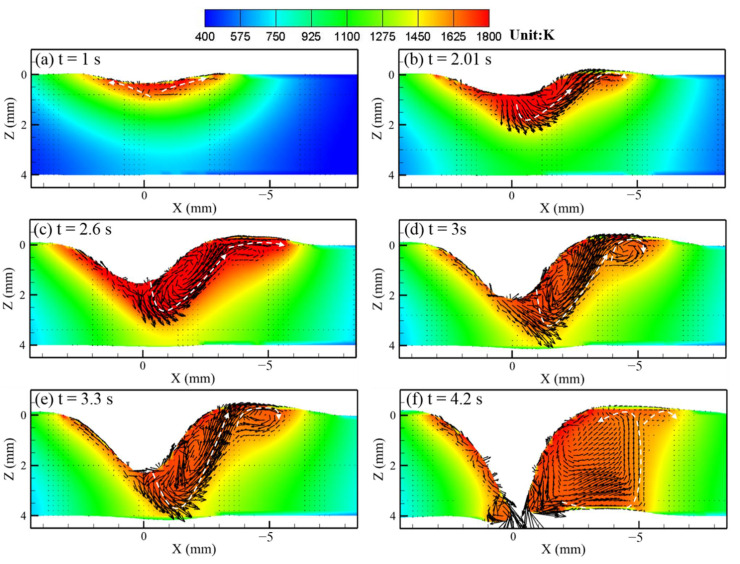
The calculated temperature field, fluid flow, and keyholing time in the U-PAW case.

Figure 9 and Figure 10 show the fluid flow and heat transfer in the weld pool during the dynamic keyhole evolution in PAW and U-PAW, respectively. The main driving forces of the molten metal flow include the Marangoni shear force caused by the surface tension gradient, gas shear stress, arc pressure, electromagnetic force, and buoyancy [18,19]. Figure 9 shows the temperature and fluid flow fields at the longitudinal cross-section in PAW. At an instant of 1.21 s, the workpiece is melted to form a shallow and little weld pool under the thermal action of the plasma arc. At this time, the surface of the weld pool is a little bit depressed. At 2.02 s, a bigger depression of the weld pool surface is caused by the plasma arc pressure, forming a shallow blind keyhole, and the molten metal flows to the rear of the weld pool. At t = 4.01 s, since the plasma arc heat and pressure on the keyhole wall reaches equilibrium, the keyhole depth reaches a value of about 2.5 mm, and does not change any more. Under this PAW condition, an open keyhole cannot be formed, but the bottom of the weld pool reaches the melting point. In Figure 9e, there are two flow circulations in the weld pool. Under the action of gas shear stress and Marangoni force, liquid metal flows from the central high temperature zone near the pool center to the low temperature zone at the rear part of the pool, forming a clockwise circulation at the rear-upper part of the weld pool. Under the action of the arc pressure and electromagnetic force, the liquid metal is driven to flow to the bottom of the weld pool, and a counterclockwise circulation is formed in most of the weld pool parts, which is consistent with the X-ray observation results in [36].

In U-PAW, due to the exerted ultrasonic vibration, the plasma jet velocity increases, and both the gas shear stress and arc pressure increase. Therefore, the ultrasonic-assisted plasma arc has a stronger keyholing capability. As shown in Figure 10, although the fluid flow trend in U-PAW is almost the same as that in PAW, an open keyhole is formed at an instant of 4.2 s. Figure 10f demonstrates that after the keyhole is formed, the liquid metal at the front keyhole wall is very thin, while there is more liquid metal on the rear wall of the keyhole. The computed results about whether the open keyhole is formed or not and the establishment time of the open keyhole are in agreement with the experimental results.

**Figure 11:**
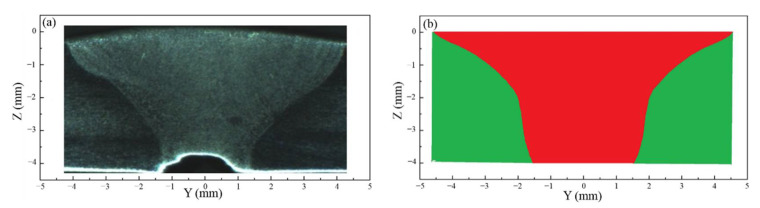
Weld cross section of U-PAW (**a**) measured (**b**) calculated.

Figure 11 shows the comparison of the experimental and calculated weld cross section in U-PAW. It can be seen that the calculated weld profile agree with the experimentally measured one. Table 3 lists the detailed comparison between the numerical calculation and experimental results of the weld cross section and keyhole exit size in U-PAW. The calculated weld widths at the top and bottom surfaces are in good agreement with the measured ones, but there is still a deviation of the fusion line track along the workpiece thickness direction. Similarly, the calculated keyhole exit size is smaller than the measured value. In our previous research, the influence of the gas shear force on the weld pool flow was neglected [22]. Therefore, the weld width at the top side is 8 mm, which is narrower than the experimental results. Considering the gas shear force, the top side weld width is closer to the experimental results. In this paper, we consider the distribution of gas shear stress on the keyhole wall through a simplified mathematical model. In the future work, we will get the distribution of the gas shear stress on the anode surface by modeling the plasma arc. In a next step, we will further improve the model and improve the numerical calculation accuracy.

## 7. Conclusions

(1) The experimental results show that under the same welding process parameters, an open keyhole can be formed after applying the ultrasonic vibration, which further shows that U-PAW improves the keyholing ability.
(2) The acoustic radiation force is considered to modify the formula of the plasma arc pressure in the ultrasonic-assisted PAW, and the prediction accuracy of the plasma arc pressure on the anode surface in U-PAW is improved.
(3) With the modified velocity of the plasma arc near the anode surface, a formula is proposed to calculate the gas shear stress on the workpiece surface.
(4) The effects of ultrasonic vibration on the plasma arc pressure and gas shear stress are considered in modeling the weld pool and keyhole behaviors in U-PAW. The dynamic keyhole evolution behavior in the weld pool is quantitatively analyzed.
(5) The numerical simulation results of the weld pool widths, establishment time of open keyhole, and keyhole exit sizes are in agreement with the experimental ones.

## Figures and Tables

**Table 1:** The used welding parameters.

Parameters	Value
Density of Ar (kg/m^3^)	1.784
Adjusting constant (N)	1.256 × 10^−6^
Plasma gas flow rate (L/min)	2.8
Conversion coefficient	2.04 × 10^−4^
Nozzle diameter (mm)	3.2
Distance from electrode to workpiece (m)	0.005
Shielding gas flow rate (L/min)	20
Sound velocity of argon (m/s)	341
Adjusting constant (N)	4.58 × 10^−4^
Vibration frequency (KHz)	25
Vibration amplitude (μm)	20
Nozzle exit area (m^2^)	8.0384 × 10^−6^

**Table 2:** Simulation cases.

	Welding Current (A)	Welding Speed (mm/min)	Ultrasonic Vibration	Workpiece Thickness (mm)
PAW	80	100	No	4
U-PAW	80	100	Yes	4

**Table 3:** Comparison of the experimental and calculated results.

Item	Measured	Calculated
Keyholing time (s)	4.6	4.2
Keyhole exit length (mm)	0.98	0.9
Keyhole exit width (mm)	1.2	0.8
Weld width (top) (mm)	8.8	8.9
Weld width (bottom) (mm)	2.8	3

## Data Availability

The data presented in this study are available on request from the corresponding author.
